# Tackling Neuroinflammation After Traumatic Brain Injury: Complement Inhibition as a Therapy for Secondary Injury

**DOI:** 10.1007/s13311-022-01306-8

**Published:** 2022-10-12

**Authors:** Inge A. M. van Erp, Iliana Michailidou, Thomas A. van Essen, Mathieu van der Jagt, Wouter Moojen, Wilco C. Peul, Frank Baas, Kees Fluiter

**Affiliations:** 1 University Neurosurgical Center Holland, Leiden University Medical Center, Haaglanden Medical Center and HaGa Hospital, Leiden and The Hague, Albinusdreef 2, J-11-R-83, 2333 ZA Leiden, The Netherlands; 2 Department of Clinical Genetics, Leiden University Medical Center, Leiden, The Netherlands; 3 Department of Intensive Care Adults, Erasmus MC – University Medical Center, Rotterdam, The Netherlands

**Keywords:** Traumatic brain injury, Neuroinflammation, Complement system, Inhibition, Narrative review

## Abstract

Traumatic brain injury (TBI) is a leading cause of mortality, sensorimotor morbidity, and neurocognitive disability. Neuroinflammation is one of the key drivers causing secondary brain injury after TBI. Therefore, attenuation of the inflammatory response is a potential therapeutic goal. This review summarizes the most important neuroinflammatory pathophysiology resulting from TBI and the clinical trials performed to attenuate neuroinflammation. Studies show that non-selective attenuation of the inflammatory response, in the early phase after TBI, might be detrimental and that there is a gap in the literature regarding pharmacological trials targeting specific pathways. The complement system and its crosstalk with the coagulation system play an important role in the pathophysiology of secondary brain injury after TBI. Therefore, regaining control over the complement cascades by inhibiting overshooting activation might constitute useful therapy. Activation of the complement cascade is an early component of neuroinflammation, making it a potential target to mitigate neuroinflammation in TBI. Therefore, we have described pathophysiological aspects of complement inhibition and summarized animal studies targeting the complement system in TBI. We also present the first clinical trial aimed at inhibition of complement activation in the early days after brain injury to reduce the risk of morbidity and mortality following severe TBI.

**Supplementary Information:**

The online version contains supplementary material available at 10.1007/s13311-022-01306-8.

## Background

Traumatic brain injury (TBI) presents a great challenge to public health worldwide. TBI is responsible for over a third of all traumatic deaths, and each year, 80–90.000 new cases of long-term disability due to TBI occur in the USA [1]. The dynamic pathophysiology that evolves over time after trauma to the central nervous system (CNS), consisting of primary injury by the direct traumatic impact, followed by secondary brain tissue injury driven substantially by host responses, makes it a highly complex problem to tackle compared to trauma in other organs [1]. Primary damage develops due to direct and contrecoup mechanical forces on the brain, including damage to neurons, axons, and glial cells and shearing of blood vessels causing hemorrhage [2]. The damage causes a breakdown of the blood–brain barrier (BBB), and changes in blood supply result in mitochondrial and subsequent energy production impairment, release of neurotransmitters and free radicals, immune cell activation and infiltration, apoptosis, and cytokine release [2, 3]. This is the initiation of the secondary brain-injury period, which occurs after a latency interval of minutes to several hours, and is probably the most important to focus on. Neuroinflammation develops over hours to days after the trauma and results in edema formation and subsequent increased intracranial pressure (ICP). Increased ICP causes additional impairment of cerebral blood flow and oxygen delivery and may contribute to brain herniation, requiring additional neuro-interventions that complicate the hospital course and final recovery [4].

TBI-induced neuroinflammation has been hypothesized to contribute very substantially to the pathological progression of brain injury, in addition to the primary injury itself [5, 6]. It is a complex interaction between the cellular components of the CNS (neurons, astrocytes, microglia), cytokines, and chemokines, in concert with influx of peripheral immune cells. Neuroinflammation is beneficial to promote clearance of debris and regeneration, but it can also cause collateral damage when dysregulated and excessive, leading to secondary brain injury. The fact that the intracranial space is inherently non-compliant, being enclosed by the rigid skull, with the evident advantage to primarily protect the brain, is a clear and unique disadvantage compared with trauma to other vital organs, when swelling occurs. Every organ develops edema in response to significant trauma, but when brain tissue develops edema facilitated by neuroinflammation, the rigid and protective skull bone is a barrier to allow for this swelling. Progressive brain edema and subsequent tissue swelling, that is refractory to ICP-lowering medical ICU treatments, will ultimately elicit brain ischemia due to impeded cerebral blood flow caused by high ICP, unless decompressive craniectomy (DC) is performed in the secondary injury phase after TBI, as has only recently been studied in a well-performed clinical trial [7].

After the (sub)acute phase of injury, a prolonged state of chronic inflammation may linger for years after TBI and predispose patients to develop other neurodegenerative disorders, such as dementia [8, 9]. Probably chronic traumatic encephalopathy is caused by a similar pathophysiology which has been shown to occur after recurrent mild trauma, like sport-related injuries [10].

Early attenuation of neuroinflammation is therefore considered an important target for TBI treatment, especially in the early in-hospital phase. Despite the vast amount of research performed to improve our understanding of the pathophysiology in TBI, the field has repeatedly experienced collective failures to translate research from animals to successful therapeutic application in humans [11, 12]. This review will summarize the most important drivers of neuroinflammation in TBI and previous trials aiming to attenuate these drivers. The main focus of the review will be the role of the complement system in post-traumatic neuroinflammation and future directions in research on complement inhibition.

## Neuroinflammation in the Clinical Setting

The primary effects of moderate and severe TBI include diffuse injuries such as diffuse axonal injury (DAI) and focal brain damage, such as epidural and subdural hematomas (ASDH) and intracerebral hematomas/contusions (tICH). In the first hours after head trauma, expansion of hematomas is the main threat, whereas during the following days, the pathophysiological consequences of neuroinflammation may subsequently increase ICP [13]. International guidelines recommend monitoring of ICP in all patients with severe TBI at high risk of secondary injuries and abnormalities on computed tomography (CT) [14]. Currently, invasive monitoring with an intraparenchymally placed sensor is the most reliable and most applied method to monitor ICP. If ICP can be maintained below a threshold of between 20 and 25 mm Hg with general supportive intensive care treatments, including appropriate pain control, ventilator support, careful fluid, and temperature management, this portends a better prognosis [15]. Management of ICP has evolved towards a “staircase” approach with an escalating treatment intensity, including cerebrospinal fluid (CSF) drainage, deeper sedation, hyperosmolar therapy to dehydrate the brain and prevent a rise in ICP, evacuation of hematomas by craniotomy and, in case of raised ICP refractory to medical managements, including barbiturates, and in the end: DC [13, 14]. High mortality has been related to the occurrence of increased ICP, and although a lower mortality has been reported when treating ICP with a DC, these patients will have higher rates of vegetative state and severe neurological disability [7]. Importantly, this current clinical practice has concentrated on trying to mitigate ICP to minimize the extent of secondary injury once this process has already started, rather than focused on managing neuroinflammatory pathways leading to a rise in ICP and thus to prevent secondary injury.

## Molecular Mechanism of Neuroinflammation After TBI

Cerebral ischemia and direct traumatic apoptosis after TBI lead to disruption of cerebral energy metabolism due to depletion of cellular adenosine triphosphate (ATP) stores. In this state, cells produce energy via the less efficient pathway of anaerobic glycolysis. Cell metabolic impairment is followed by membrane depolarization and release of excitatory glutamate [16]. Excess accumulation of extracellular glutamate causes neuronal excitotoxicity, which is a major contributor to post-traumatic neurodegeneration. It results in excessive calcium influx within the neuronal cytoplasm due to increased activation of the N-methyl-D-aspartate (NMDA) receptors and voltage-dependent ion channels [17]. The increased intracellular Ca^2+^ levels lead to mitochondrial damage, lipid peroxidation, production of free radicals, and activation of caspases and other proteases involved in membrane and nucleosomal DNA changes. This results in synapse elimination and neuronal apoptosis [17, 18].

Cell injury in the brain results in the release of intracellular components such as ATP, other damage-associated molecular patterns (DAMPs), and complement components which activate pattern recognition receptors (PRRs) on glial cells. As such, injury of brain cells initiates and perpetuates a post-traumatic neuroinflammatory response [19, 20].

**Fig. 1:**
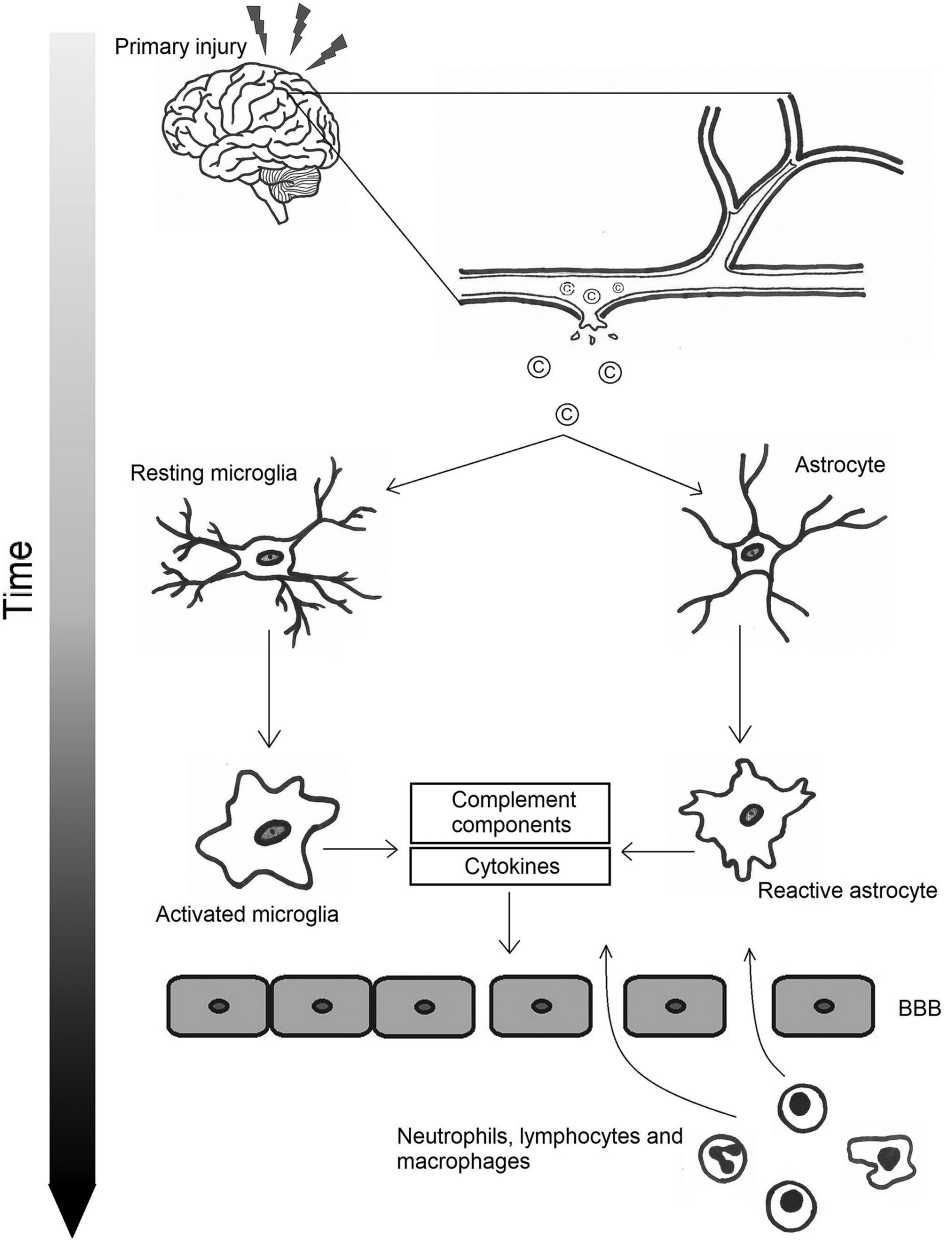
Pathophysiology of neuroinflammation after TBI

Microglia are brain cells acting as support cells for neurons and other metabolic and immunological processes and play a critical role in neuroinflammation as the first line of defense. Microglial cell activation is prominent at the perilesional area and in ipsilateral and contralateral regions of the contusion area [21]. In the post-traumatic phase, microglial cells undergo morphological transformations and changes in their gene expression repertoire to produce a wide spectrum of pro- or anti-inflammatory cytokines (Fig. 1). A proinflammatory M1 phenotype characterized by the production of IL-1β and TNF-α, nitric oxide and reactive oxygen species (ROS), and an anti-inflammatory M2 phenotype characterized by the production of IL-10, IL-13 and transforming growth factor (TGF)-β have been previously described for the activated microglia [22]. However, nowadays is it well recognized that the M1 and M2 states are the extremes of a continuum of activation states and intermediate phenotypes are present [23]. In TBI, microglial polarization is being skewed towards a proinflammatory state varying with increasing time after the trauma [24]. Similar to microglia, a proinflammatory and an anti-inflammatory state have been described for activated astrocytes [25]. These proinflammatory astrocytes secrete many cytokines and other factors that may greatly enhance the inflammatory response [25]. Astrocytes may respond to trauma with proliferation followed by assembly of a dense barrier, known as the glial scar, aiming to protect healthy tissue from nearby areas of neuroinflammation [26].

Important factors in the promotion of neuroinflammation are proinflammatory cytokines. Well known is IL-1β, involved in oligodendrocyte damage and early microglia activation. Levels of IL-1β correlate with Glasgow Coma Scale (GCS) scores, ICP, and outcome [27, 28]. Production of IL-1β by glia requires an activated NLRP3 inflammasome, which catalyzes the cleavage of pro-interleukins into their active forms [29]. Deactivation of inflammasomes results in the alleviation of brain edema, reduction of lesion volume, and improvement of long-term motor and cognitive function in experimental TBI in animals [30, 31]. TNFα is another key cytokine in post-traumatic cerebral neuroinflammation. Human carriers of two TNF alleles, resulting in higher TNFα production in response to TBI, had a higher probability of poor outcome after TBI [32, 33].

Besides their role in neuroinflammation, proinflammatory cytokines may also challenge the integrity of the BBB vasculature. This could lead to vasogenic brain edema and penetration of serum proteins into brain interstitium, such as complement components and fibrin which can further activate glial cells [34]. The BBB consists of endothelial cells, astrocytic endfeet, and pericytes, and its integrity results from the selectivity of the tight junctions between the endothelial cells to restrict the passage of solutes [35]. Disruption of the BBB integrity is primarily caused by damage of these tight junction proteins, especially occludin and claudin-5, and it is further challenged by post-traumatic systemic inflammation which promotes leukocyte chemotaxis and transendothelial migration [36, 37].

Leukocyte recruitment into the CNS is mediated by upregulation of endothelial and leukocyte adhesion molecules and activated complement fragments, as discussed later on [38]. Neutrophils are the first-line transmigrated immune cells and can be found as early as 2 h after injury and peak within 24–48 h, before rapidly declining over the following days [39]. Cerebral accumulation of neutrophils has been associated with increased secondary brain damage and adverse outcome [40]. The early neutrophil recruitment is followed by infiltration of lymphocytes and monocyte-derived macrophages. Of note, the neutrophil-to-lymphocyte ratio has been reported as an objective, low-cost, and early predictor of inflammation and clinical outcome in TBI patients [41].

## The Complement System in TBI—Pathophysiology and Animal Models

**Fig. 2:**
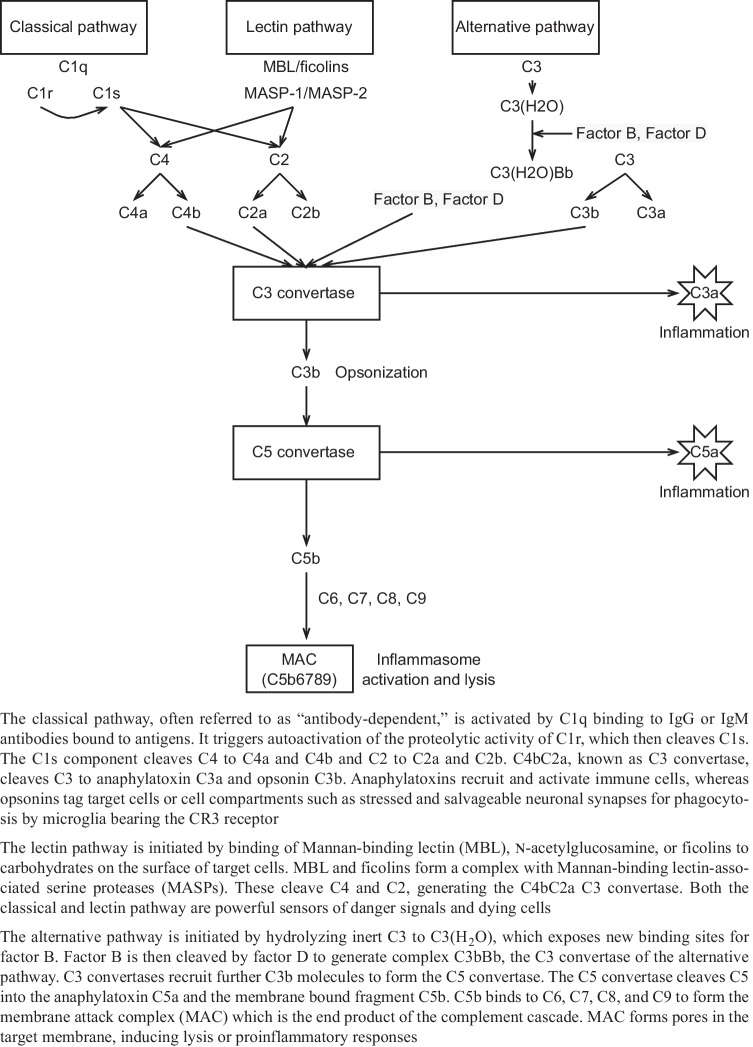
Pathways of the complement system

The complement system functions to eliminate foreign pathogens and substances and remove debris and apoptotic cells [42]. Complement activation can be mediated in three distinct pathways: the classical, lectin, and alternative pathways (Fig. 2), all resulting in the formation of the membrane attack complex (MAC). The host is protected against overactivation of the complement cascade by the complement regulatory proteins. These include the C1-inhibitor (C1-INH), which inactivates the C1r, C1s, and MASPs, the decay-accelerating factor (DAF or CD55) which accelerates the breakdown of C3 and C5 convertases, factor H (fH) leading to breakdown of the C3 convertase, membrane cofactor protein (MCP or CD46) aimed to cleave C3b, and CD59 protein which prevents MAC formation.

Complement has a key contribution to pathophysiological events that sequester the initial impact of injury and may radiate damage from the contusion core to the penumbra after TBI. For instance, glial synthesis of complement C3 and clusterin, a regulator of complement, was found in the vicinity of experimental brain contusion [43]. Further, C3d and C9 were localized on neurons in the vicinity of experimental brain contusion, suggesting that these neurons are targets of complement proteins [43].

High-quality preclinical evidence suggests that inhibition of various complement factors can improve neurological performance and reduce inflammation (Table 1). For example, our data on the closed head injury model of TBI showed that pharmacological inhibition of MAC formation improved neurological performance in mice by reducing inflammasome activation and preventing microglial activation and axonal damage [44]. In line with these data, mice with a genetic deletion in the CD59a gene, being deficient for a major regulator of MAC formation and therefore resulting in excessive MAC formation, showed increased neuronal cell death and brain tissue destruction following head trauma [45, 46].

**Table 1:** Summary of preclinical studies aiming to inhibit the complement system after TBI

**Author (year)**	**Species**	**Model**	**Groups**		**Pathway**	**Main findings**	**Ref**
			**Treatment**	**Control**			
**Alawieh et al. (2018)**	Mice	Controlled cortical impact	CR2Crry / CR-2fH / CR2CD59	WT mice	All / alternative / terminal	Inhibition of C3 activation, but not of MAC formation, achieved sustained neuroprotection. Furthermore, the fact that CR2Crry and CR2fH provided similar levels of protection indicates an important role for the alternative pathway in TBI	[94]
**De Blasio et al. (2017)**	Mice	Controlled cortical impact	MBL -/- mice	Saline (WT or MBL -/- mice)	Lectin	Selective pharmacological inhibition of MBL, and, in particular of the MBL-C isoform, improves functional neurobehavioral outcome following TBI	[105]
**Fluiter et al. (2014)**	Mice	Weight drop model	C6 antisense oligonucleotide / C5-binding protein	Saline	Terminal	Specific inhibition of the MAC reduces neuronal apoptosis and axonal loss and promotes recover of neurologic performance after TBI	[44]
**Hicks et al. (2002)**	Rats	Lateral fluid percussion	Vaccinia virus complement control protein (VCP)	Saline	Classic and alternative	VCP attenuates impairments in spatial memory, but not neuropathological damage, as compared to the saline treated controls	[106]
**Kaczorowski et al. (1995)**	Rats	Weight drop model	sCR1	Saline	Classic	Neutrophil accumulation occurring in the brain after trauma is inhibited by sCR1 treatment	[107]
**Kotwal et al. (2002)**	Rats	Fluid percussion	VCP	Saline	Classic and alternative	VCP can improve cognitive deficits following TBI	[108]
**Krukowski et al. (2018)**	Mice	Controlled cortical impact	Genetic: C3-/- Pharmacological: anti-C1q antibody	Genetic: WT mice Pharmacological: saline	Classic	Inhibition of the classical complement cascade, either through deletion of C3 or inhibition of C1q, provides protection against cognitive decline	[109]
**Leinhase et al. (2007)**	Mice	Weight drop model	mAb1379 monoclonal anti-factor B antibody	Saline (PBS)	Alternative	Systemic administration of an inhibitory anti-factor B antibody led to a substantial attenuation of cerebral tissue damage and neuronal cell death. No difference was found in neurological outcome between groups	[110]
**Leinhase et al. (2006)**	Mice	Weight drop model	fB -/- mice	WT mice	Alternative	The alternative pathway of complement activation plays a major role in contributing to the overall extent of post-traumatic complement activation (C5a generation) and to neuronal cell death after brain injury	[62]
**Leinhase et al. (2006)**	Mice	Weight drop model	Crry-Ig	Saline (PBS)	Classic and alternative	Post-traumatic pharmacological blocking of complement activation by Crry-Ig leads to significantly increased neurological recovery after head injury, protection of neuronal subsets, and upregulation of complement-regulatory genes and anti-apoptotic mediators	[111]
**Li et al. (2013)**	Rats	Moderate blast overpressure	rhDAF	Saline	Classic and alternative	DAF suppresses the systemic and local inflammatory response, reduces tau phosphorylation, improves BBB integrity, and decreases cytotoxic edema	[112]
**Longhi et al. (2014)**	Mice	Controlled cortical impact	MBL-A and MBL-C -/- mice	WT mice	Lectin	MBL deficiency in mice subjected to TBI is associated with long-term attenuated post-traumatic functional deficits and tissue damage	[80]
**Longhi et al. (2009)**	Mice	Controlled cortical impact	C1-INH	Saline	Classic	Administration of C1-INH attenuates neurobehavioral deficits and histological damage and reduces contusion volume after TBI	[98]
**Mercurio et al. (2020)**	Mice	Controlled cortical impact	Masp2-/-, Fcna-/-, Mbl-/- mice	WT mice	Lectin	MASP-2-/-, MBL -/-, and FCN-A-/- mice had lower neurological deficits and higher neuronal density after TBI. The highest degree of protection is achieved through the absence of the LP key enzyme MASP-2	[113]
**Neher et al. (2014)**	Mice	Weight drop model	CR2-/- mice	WT mice	Classic and alternative	Head injured CR2-/- mice displayed a significantly improved neurological outcome, significantly reduced post-traumatic mortality, attenuated extent of neuronal cell death and of post-injury astrogliosis, and reduced intracerebral complement C3	[114]
**Pillay et al. (2007)**	Rats	Lateral fluid percussion	VCP	Saline	Classic and alternative	VCP administration significantly influenced sensorimotor function recovery, but did not significantly improve the cognitive outcome after severe head trauma	[115]
**Pillay et al. (2005)**	Rats	Fluid percussion	VCP	Saline	Classic and alternative	In a mild injury model, VCP influences neurologic outcome and offers some enhancement in spatial memory and learning	[116]
**Rancan et al. (2003)**	Mice	Weight drop model	GFAP-sCrry transgenic mice	WT mice	Classic and alternative	Transgenic mice with astrocyte-targeted expression of the soluble complement inhibitor sCrry have a significantly reduced neurologic impairment and improved blood–brain barrier function after closed head injury	[117]
**Rich et al. (2016)**	Mice	Weight drop model	mTT30 (CR2-fH)	Saline (PBS)	Alternative	mTT30 attenuated complement C3 deposition in injured brains, reduced the extent of neuronal cell death, and decreased post-injury microglial activation	[118]
**Ruseva et al. (2015)**	Mice	Weight drop model	CD59-2a-CRIg	WT mice	Terminal	MAC inhibition results in reduced inflammation, neuronal stress, and enhanced neurologic recovery	[46]
**Sewell et al. (2004)**	Mice	Cryoinjury	C5a receptor antagonist	WT mice	All	Inhibition of C5 accounts for the majority, but not all, of the neutrophil extravasation in TBI	[61]
**Stahel et al. (2009)**	Mice	Weight drop model	CD59a-/- mice	WT mice	Terminal	Absence of regulatory molecule CD59a results in impaired neurological outcome	[45]
**Weiss et al. (2020)**	Sprague Dawley rats	Weight drop model	C1-INH	N/A	Classic	Inhibition of C1 results in inhibition of complement activation and reduces edema formation	[99]
**Yager et al. (2008)**	Mice	Controlled cortical impact	MBL -/- mice	WT mice	Lectin	Absence of MBL results in decreased cognitive brain function	[119]
**You et al. (2007)**	Mice	Controlled cortical impact	C4-/- mice	WT mice	Classic	Inhibition of C4 improves recovery and post-traumatic motor deficits and reduces overall brain tissue damage (independent of C3 activation)	[63]

*BBB* blood–brain barrier, *MAC* membrane attack complex, *MASP* Mannan-binding lectin-associated serine protease, *MBL* Mannan-binding lectin, *VCP* Vaccinia virus complement control protein, *WT* wild type

Primary injury results in brain tissue destruction, including shearing of blood vessels causing hemorrhage. Within the CNS, complement factors are activated, resulting in activation of both microglia and astrocytes (to M1 and A1 phenotype). Activation of microglia and astrocytes leads to activation of more complement factors and cytokines. Finally, disruption of the blood–brain barrier arises, causing infiltration of neutrophils, lymphocytes, and macrophages from the peripheral hemodynamic system to the CNS interstitium

Facilitation of phagocytosis is another important contribution of complement activation to secondary damage in TBI. Cerebral biosynthesis of most complement proteins is induced in response to trauma and adds to the pool of complement proteins which penetrate the compromised BBB [47]. Intracerebral complement activation leads to the generation of complement opsonins which facilitate the clearance of debris at the site of injury by microglia and macrophages bearing the CR3 receptor [48]. In addition, elevated levels of C1q within the cerebral parenchyma promote the transformation of microglia to the proinflammatory phenotype [49]. Lastly, C1q and C3 play a key role in microglia-mediated synapse elimination which is a prominent neurodegenerative mechanism after head trauma [50].

Activated microglial cells induce a proinflammatory phenotype to astrocytes via secretion of C1q, IL-1α, and TNFα, contributing to the rapid death or functional disability of neurons and oligodendrocytes [25]. Proinflammatory astrocytes, in turn, secrete many complement components, such as C3, that enhance the neuroinflammatory response [25, 51].

In addition to the complement components which are synthesized by neurons and glial cells within the CNS [52, 53], there is an influx of complement factors from the blood due to breakdown of the BBB after TBI [54, 55]. The anaphylatoxins C3a and C5a act as chemoattractants for immune cells expressing the relevant receptors such as the granulocytes which are present within TBI lesions [56]. In addition, in vitro studies showed that C5a activates the expression of beta2-integrin on neutrophils promoting their adhesion to the inflamed endothelium of the BBB and stimulates the secretion of the proinflammatory mediators TNFα and IL1 by human mononuclear cells [57–59]. Notably, animal studies showed that genetic deletion of the genes encoding for the C3 or the C5 component or pharmacological inhibition of C3 or C5 resulted in a reduction of neutrophil infiltration, injury size, microglial activation. and brain edema leading to significantly improved neurological outcomes [60–64].

## The Interaction of the Complement System with the Coagulation System

In TBI, hemostasis is often derailed, either leading to a hypo-coagulopathic state on one end of the spectrum, causing cerebral bleeding disorders leading to progression of contusions into growing t-ICHs and ASDHs, and to a hyper-coagulopathic state that contributes to ischemic lesions due to (micro)vascular thrombosis in lesioned areas [65]. The intimate interaction and co-evolution of the coagulation system together with the complement system is widely appreciated within the basic science research field. The complement system has been found to increase tissue factor activity, thereby activating the extrinsic coagulation pathway, and form activated thrombin from prothrombin. Moreover, complement factors increase platelet activity and aggregation and prothrombinase activity, including von Willebrand factor and P-selectin. Classical and lectin pathway activation has been reported to be associated with increased odds of venous thromboembolism in the clinical setting [66, 67]. Moreover, in sepsis patients, disseminated intravascular coagulation (DIC) was correlated to the degree of complement activation [68]. It is also known that MAC attenuates endothelium-dependent relaxation leading to a hypertensive state [69]. This evidence, reviewed in [70], suggests that complement overactivation shortly after TBI could potentially lead to increased coagulation activity, i.e., a prothrombotic state often seen days to weeks following TBI. Nevertheless, the correlation between trajectories of complement activity and markers of hemostasis and platelet function, specifically in TBI, warrants more research.

## Complement Activation in TBI—Human Studies

In TBI patients, high levels of C4, C3, and MAC have been found in serum [54, 71–76], and upregulation of factor B, C3, and MAC was detected in the CSF of severe TBI patients [55, 77]. Moreover, increased immunoreactivity was found in resected contused tissue for C1q, C3b, C3d, and MAC within/on neurons located in the penumbra area [78, 79]. Intracerebral deposition of MBL, ficolin-2 and 3, and MASP-2 and 3 was found after TBI within the vasculature and in the injured perivascular tissue [79, 80]. High levels of complement proteins were strongly associated with lower GCS scores and independently predict mortality or unfavorable clinical outcomes in TBI [73, 75]. A proteomics study using human frontotemporal cortex samples showed a consistent overexpression of C4a, C4b, C3, C7, and C9 [81]. More recently, microvesicles and exosomes were analyzed in the CSF of TBI patients and mass spectrometry-based proteomic identification of proteins indicated presence of complement C1q [82]. Furthermore, plasma astrocyte-derived exosomes (ADEs) protein levels of C4b, factor D, Bb, MBL, C3b, and MAC were significantly higher and those of the regulatory proteins CR1 and CD59 lower in the first week of TBI compared to controls [83].

The complement system is further triggered by secondary insults [84]. Expression of complement proteins C3, C8a, and C9 is still increased in the plasma of TBI patients at 1, 3, and 6 months after injury compared to controls, suggesting persistent complement activation during the subacute and chronic phase [85]. Prolonged complement activation has been linked to early-onset cognitive decline, behavior disorders, and predisposition to dementia syndromes like Alzheimer’s disease [86].

## Clinical Trials to Control Neuroinflammation: What Has Been Tried So Far?

Studies on the dysregulated inflammatory response are important to serve as a roadmap for future clinical trials aiming at “targeted” pharmacological neuroprotection and improved neurological recovery after TBI. Although all of the described interventions within trials up to now (Table 2) have shown to be effective in preclinical and small single center phase II trials, successful translation to phase III clinical trials showing efficacy of these treatments has not yet been accomplished. Multiple explanations have been proposed to explain these failures. First, the heterogeneity of the TBI population and the large treatment variation in the management of TBI indicate that large sample sizes are warranted to achieve any statistical significant difference between groups [87, 88]. Second, there is a growing recognition of the problem of age and sex bias on the outcomes in neurotrauma research, as for example, fewer women than men are recruited in clinical trials (Table 2) [89, 90]. Third, most trials focused on “delayed” outcome metrics, such as the Glasgow Outcome Scale Extended (GOSE) at 6 months, as primary endpoint, whereas animal models focus on the direct impact of a therapy on microglial activation, edema formation, or neuronal death.

**Table 2:** Summary of randomized controlled trials aiming to attenuate neuroinflammation after TBI

**Author (year)**	**Study design**	**Patients: N(GCS)**	**Age, years**	**% male**	**Intervention**	**Main outcome**	**Conclusion and remarks**	**Ref**
**Steroids** *Reduction in apoptosis, microglial activation, cytokines, and enhanced production of CD55 to reduce complement activation* [120–124]								
**Skolnick et al. (2014)**	‘SYNAPSE’ Multicenter phase III trial	1195 (GCS ≤ 8)	35 [IQR 23–51]	78	Progesterone LD 0.71 mg/kg per hour for 1 h, 0.50 mg per kg per hour for 119 h or placebo	No difference in GOS-E 6 months	No evidence to support progesterone in TBI	[125]
**Wright et al. (2014)**	‘PROTECT’ Multicenter phase III trial	882 (GCS 4–12)	35 [range 17–94]	74	Progesterone LD 14.3 ml per hour for 1 h, 10 ml per hour for 71 h to a total of 96 h or placebo	No difference in GOS-E 6 months	No evidence to support progesterone in TBI; early termination due to futility analysis	[126]
**Edwards et al. (2005)**	‘CRASH’ Multicenter phase III trial	10,008 (GCS ≤ 14)	37 (SD 17)	81	48 h methyl-prednisolone or placebo	Higher mortality risk at 2 weeks in steroid group	Nonspecific, high-dose immune suppression is detrimental	[91]
**Bradykinin antagonist** *Blocks bradykinin to reduce immune cell influx, ICP, and contusion volume and improve neurological outcome* [127–131]								
**Shakur et al. (2009)**	‘BRAIN trial’ Multicenter phase II trial	228 (GCS ≤ 12)	36 (SD 14)	89	Low (10 mg LD and 5 mg/day), medium (20 mg LD and 10 mg/day), or high (30 mg LD and 15 mg/day) dose anatibant or placebo	No differences in SAEs, mortality, GCS, DRS, and HIREOS	Early termination due to funding withdrawal resulting in an underpowered study; still no reliable evidence	[132]
**Cyclosporin A** *Selective inhibition of T-cell-mediated immune response and reduction of acute damage after TBI* [133]								
**Mazzeo et al. (2009)**	Dual-center phase II trial	50 (GCS ≤ 8)	31 (SD 15)	82	5 mg/kg cyclosporin A over 24 h or placebo	No differences in BUN, creatinine, HB, PLT, WBC or AEs	Excellent safety profile but no significant differences due to low sample size	[134]
**Cannabinoid** *Inhibits production of the proinflammatory cytokine TNFα, reduces BBB breakdown, attenuated the development of cerebral edema and accumulation of calcium, and improves the short and long term recovery of motor and memory functions* [135–137]								
**Maas et al. (2006)**	Multicenter phase III trial	861 (GCS motor 2–5 and ICP monitoring)	33 [IQR 23–46]	82	150 mg dexanabinol or placebo	No difference in GOS-E 6 months	No evidence to support dexanabinol in TBI	[138]
**Knoller et al. (2002)**	Multicenter phase II trial	67 (GCS 4–8)	30 (SD 13)	85	48 or 150 mg dexanabinol or 1of 2 ml placebo	Reduction in time of high ICP, low CPP and low SBP, higher GOS at 3 and 6 months	Safe, well tolerated and a better ICP/CPP control without jeopardizing BP with a better neurological outcome	[139]
**Erythropoietin** *Decrease of IL-1β, TNFα, CCL-2, macrophage-inflammation protein (MIP)-2, and intercellular adhesion molecule (ICAM)-1* [140–142] *Reduction of BBB permeability and apoptotic cells with attenuation of brain edema and improved cognitive function in TBI* [142–145]								
**Nichol et al. (2015)**	‘EPO-TBI’ Multicenter phase III trial	606 (GCS ≤ 12)	31 [IQR 23–48]	84	40,000 UI erythropoietin or placebo one a week for maximum of three doses	No difference in GOSE 6 months	They did find a reduced mortality in patients without lesions and in adjusted analysis with extracranial injury. Underpowered study but potential target [146]	[147]
**Robertson et al. (2014)**	Multicenter phase III trial	200 (closed head injury)	30 [IQR 22–47)	86	5000 UI/kg per dose erythropoietin or placebo	No difference in GOS 3 months	No evidence to support EPO in TBI	[148]
**Interleukin 1-antagonist** *Blocks IL-1 signal transduction to attenuate microglial activation, TNFα production, and infiltration of neutrophils and T-cells* [27, 149, 150]*. It results in reduced lesion volume, hemispheric tissue loss, and attenuated cognitive deficits* [151, 152]								
**Helmy et al. (2014)**	Single center, open label, phase II trial	20 (GCS ≤ 8)	39 [range 18–61]	50	100 mg interleukin-1 receptor antagonist (IL1ra) once a day for 5 days or placebo	Increase in IL-1a in brain extracellular space and circulation	IL1ra is able to cross the BBB. A subsequent study showed the IL1ra group to have cytokines biasing to M1-like microglial phenotype indicating a pro-inflammatory milieu [153]	[154]
**Hypothermia** *Decreases inflammasome signalling in neurons and reduces the innate immune response* [155, 156]								
**Cooper et al. (2018)**	‘POLAR’ Multicenter phase III trial	511 (GCS ≤ 8)	35 (SD 14)	80	Cooled in out-of-hospital and ED for at least 72 h and up to 7 days or normothermia	No difference in GOS-E 6 months	No evidence to support hypothermia as primary strategy in TBI	[157]
**Clifton et al. (2011)**	‘NABIS: H II’ Multicenter phase II trial	232 (GCS ≤ 8)	28 (SD 10)	Not reported	Cooled to 33 °C for 48 h and then gradually rewarded or normothermia	No difference in GOS-E 6 months	No evidence to support hypothermia as primary strategy in TBI. Early termination due to futility analysis but a post hoc analysis reported improved outcome of patients with hypothermia before or soon after craniotomy [158]	[159]
**Metformin** *Inhibits microglial activation and decreased production of TNFα, IL-1β, and IL-6 through NF- κB inhibition* [160–163]								
**Taheri et al. (2019)**	Single center, phase II trial	30 (GCS ≤ 8)	31 (range 19–61)	100	2 g metformin every 12 h for 5 days or placebo	Decline in S100b and NLR values toward normal levels in the metformin group	Excellent safety profile, could potentially be an effective intervention in TBI	[164]
**Statin** *Reduction of IL-1β, IL-6, IFN-ƴ, TNFα, activation of microglial cells and astrocytes, and invasion of T cells, neutrophils, and natural killer (NK) cells* [165, 166]*. Results in change of immunomodulatory profile by both inhibiting M1 polarization and enhancing M2 polarization* [166]								
**Farzanegan et al. (2017)**	Single center, phase II trial	65 (GCS 5–13)	33 (SD 17)	91	20 mg atorvastatin or placebo for 10 days	No difference in contusion volume and expansion	GOS, mRS, and DRS at 3 month follow-up were significantly better in the treatment group. A large trial is warranted	[167]
**Sanchez-Aguilar et al. (2013)**	Single center, phase II trial	36 (GCS ≤ 12)	25 (IQR 19–31)	94	20 mg rosuvastatin or placebo for 10 days	Reduction of TNFα but no reduction of IL-1β, IL-6, and IL-10 with treatment	The treatment was associated with a more favorable functional outcome. Statins may have an anti-inflammatory effect that promotes recovery	[168]
**Hypertonic saline** *Decreases aquaporin 4, TNFα, and IL-1β mRNA and protein levels* [169, 170]								
**Bulger et al. (2010)**	Multicenter, phase III trial	1331 (GCS ≤ 8)	39 (SD 18)	76	Single 250 ml bolus of 7.5% saline/6% dextran 70 vs. 7.5% saline vs. 0.9% saline	No difference in GOS-E 6 months	No evidence to support hypertonic saline as primary strategy in TBI	[171]
**Minocycline** *Inhibits the excitotoxic NMDA pathway, reduces pre-apoptotic caspase activity and IL-1β and TNFα production, and improves microglial activity* [172–174]								
**Meythaler et al. (2019)**	Single center, phase IIa trial	15 (GCS 3–12)	43 [range 21–71]	80	Minocycline LD 800 mg 200 mg BID 7 days or LD 800 mg 400 mg BID 7 days or placebo	No difference in adverse events	A trend towards an improved outcome was observed. Large trials are required to access minocycline in TBI	[175]

*BBB* blood–brain barrier, *GCS* Glasgow Coma Scale, *GOS-E* Glasgow Outcome Scale Extended, *ICP* intracranial pressure, *CPP* cerebral perfusion pressure, *SBP* systolic blood pressure, *NLR* neutrophil to lymphocyte ratio, *LD* loading dose, *BID* twice a day

Last and most importantly, most trials did not focus on a targeted dysregulated part of neuroinflammation after the first TBI impact. The steroid trials, as described in Table 2, show that broad inhibition of the immune response can be deleterious after TBI, and therefore a more targeted approach focused on a specific neuroinflammatory pathway and during a limited period of time may be more successful in improving outcomes [91, 92]. Current and future clinical trials aiming to reduce secondary brain injury should focus on targeting well-defined specific pathways with a closely related endpoint to the therapeutic mechanism of action to test efficacy. This should be based on both thorough and sufficient preclinical testing in multiple injury models (including different age ranges and sex), together with a detailed insight into the most important drivers of TBI pathophysiology for each individual patient.

## Complement Inhibition in the Clinical Setting—Future Directions

Currently, no clinical trials are present in literature aiming to inhibition complement activation in brain injury. Only a few drugs, C1-esterase inhibitors (C1-INH), Cinryze, Berinert and Ruconest, and C5-inhibitors, eculizumab and ravulizumab, are approved complement inhibitory drugs, but many others are in clinical development. The indications for the current approved drugs are hereditary angioedema (C1-INH) and paroxysmal nocturnal hemoglobinuria, atypical hemolytic uremic syndrome, and neuromyelitis optica (C5). A trial with C5-antibodies in patients with aneurysmal subarachnoid hemorrhage (SAH) is now recruiting participants [93]. As it has been reported that inhibition more upstream in the complement cascade is necessary to prevent the amplification of a feedforward mechanism of neuroinflammation that persists throughout the chronic phase [94], C1-INH might be more effective to attenuate complement overactivation. C1-INH is a potent multi-target serpin, which effectively inhibits activation of the classical, lectin, and alternative pathways [95–97]. Administration of C1-INH in animal models showed reduced contusion volume and brain water content and improvement of cognitive and motor function [98, 99]. In addition to its role in complement inhibition, C1-INH is a known inhibitor of FXIIa, FXIa, FXII, thrombin, kallikrein, HMWK prekallikrein complexes, and plasmin which inhibit fibrinolysis, contact activation, and coagulation [100, 101]. Efficacy and an excellent safety profile of high doses of C1-INH have been reported in off-label trials in sepsis and ischemia–reperfusion injury patients [102, 103]. Therefore, we are currently recruiting TBI patients in the Complement Inhibition: Attacking Overshooting inflammation @fter Traumatic Brain Injury (CIAO@TBI) trial to assess the safety and efficacy of C1-INH in this patient population [104]. In this trial, patients will be randomized to either receive one dose of 1600 IU C1-INH or a placebo injection. The primary outcome is the therapy intensity level scale that measures all ICP-directed interventions. This study will provide insight in the promising role of complement inhibition in brain injury. In the meantime, more research is warranted towards defining the inflammatory phenotypes of our patients based on injury characteristics (e.g., age, sex, and injury severity), imaging, and biomarkers to eventually being able to target inflammation with personalized immunomodulatory treatments,

## Conclusion

Neuroinflammation is one of the “nonsurgical” key drivers causing secondary brain injury after TBI. Attenuation of the inflammatory response is a potential therapeutic target. This review covers the most important neuroinflammatory drivers resulting from TBI and summarizes the clinical work performed to date directed to attenuate neuroinflammation. The complement system play an important role in the pathophysiology of TBI, and therefore therapies targeting this pathway might contribute to future targeted therapy, currently evaluated in a clinical trial.

## Supplementary Information

Below is the link to the electronic supplementary material.

Supplementary file1 (PDF 114 kb)

Supplementary file2 (PDF 508 kb)

Supplementary file3 (PDF 119 kb)

Supplementary file4 (PDF 123 kb)

Supplementary file5 (PDF 3188 kb)

Supplementary file6 (PDF 2260 kb)

Supplementary file7 (PDF 119 kb)

Supplementary file8 (PDF 435 kb)

## Data Availability

Not applicable.

## Abbreviations

C1-INH: C1-inhibitor
CNS: Central nervous system
CSF: Cerebrospinal fluid
GCS: Glasgow Coma Scale
GOSE: Glasgow Outcome Scale Extended
ICP: Intracranial pressure
TBI: Traumatic brain injury
TXA: Tranexamic acid
